# Effect of Premature Acute Coronary Syndrome on Ocular Hemodynamics

**DOI:** 10.7150/ijms.107842

**Published:** 2025-03-03

**Authors:** Jia-lin Wang, Lan-ting Wu, Yan-ling Wang

**Affiliations:** Department of Ophthalmology, Beijing Friendship Hospital, Capital Medical University, Beijing 100050, China.

**Keywords:** premature acute coronary syndrome, ophthalmic artery, computational fluid dynamics, hemodynamics, three-dimensional reconstruction

## Abstract

**Background:** Premature acute coronary syndrome (ACS) has attracted attention due to high rates of recurrent ischemic events and mortality. We aimed to explore the characteristic changes of the ocular hemodynamics in patients with premature ACS.

**Methods:** 116 participants (30 healthy controls, 30 with premature ACS, 56 with non-premature ACS) undergoing computed tomographic angiography (CTA) were enrolled. Head and neck CTA images were used to construct three-dimensional models of participants' ophthalmic arteries (OAs). Morphological parameters were measured, and numerical simulations based on computational fluid dynamics were used to acquire hemodynamic parameters of OAs. Retinal and choroidal vascular parameters were obtained by color fundus images and optical coherence tomography.

**Results:** No significant differences in morphology of the OAs among the three groups. Hemodynamic simulation showed a significantly slower OA blood velocity in patients with premature ACS than in the control (*P* < 0.001) and non-premature groups (*P* = 0.038). Lower wall shear stress was found in patients with premature ACS than that in control (*P* = 0.015) and non-premature groups (*P* = 0.049). Patients with non-premature ACS had a higher wall pressure than healthy controls (*P* = 0.035). Mass flow ratios were decreased in all ACS groups (*P* < 0.001). Patients with premature ACS had smaller central retinal artery equivalent and choroidal vascularity index. The hemodynamic parameters of OA were correlated with several clinical indicators.

**Conclusions:** Hemodynamics changes of OA and microcirculation of the retina and choroid in patients with premature ACS appeared before OA morphological changes. Premature ACS may aggravate ocular ischemic lesions more than non-premature ACS. Our findings could potentially guide future studies into a better understanding of the association of ocular lesions with systemic conditions in patients with premature ACS.

## Background

An estimated 197 million individuals worldwide are affected by ischemic heart disease, which, despite advancements in prevention and treatment, continues to lead global mortality rates^1^. Acute coronary syndrome (ACS), an acute manifestation of ischemic heart disease, is a group of diseases that includes ST-segment elevation myocardial infarction (STEMI), non-ST-segment elevation myocardial infarction (NSTEMI), and unstable angina (UA)^2^. In recent years, premature ACS, generally referred to as ACS in men ≤55 years or women ≤65 years old^3^, ^4^, has attracted much attention due to its high rates of ischemic recurrent events and mortality^5^. However, little evidence exists of the characteristics of individuals with premature ACS and its systemic impact.

The vasculatures of the heart and eye have the same histological origin, similar microcirculation anatomy, and are exposed to the same internal environment. Cardiovascular function and risk factors for cardiovascular disease interact with the occurrence and progression of many ocular diseases^6^. Park *et al.*^7^ reported a 3.7% incidence of stroke and ACS in the 6 months before central retinal artery occlusion. Therefore, it is imperative to pay attention to ocular changes in patients with ACS. However, few studies have examined ocular hemodynamic changes. Most previous studies analyzed the retrobulbar blood flow of patients with ACS using color Doppler imaging^8^ and summarized the morphological and functional characteristics of retinal vessels^9^. The ophthalmic artery (OA) is the first main branch of the internal carotid artery (ICA); hence, it is the source of direct blood supply to the eye that can reflect the ocular blood flow more directly and accurately. Nevertheless, it is difficult to observe the OA because of its narrow diameter and complex course.

Our team first used computational fluid dynamics (CFD) to investigate OA morphology and hemodynamic features in patients with ACS in a previous study^10^. Through numerical simulation, we found that the OA blood flow velocity in patients with ACS decreased significantly and was correlated with clinical parameters. The CFD-based OA numerical simulation provides a new perspective on the association between ischemic heart disease and ocular symptoms. However, ocular changes in premature ACS have not been discussed. Therefore, this study aimed to explore the changes in the OA in patients with premature ACS and further improve our understanding of the relationship between ischemic heart disease and ocular lesions.

## Methods

### Study participants and data collection

This study (ChiCTR2100050428) included patients admitted to Beijing Friendship Hospital from September 2021 to February 2022 who had undergone head and neck computed tomographic angiography (CTA) and were diagnosed with ACS, as well as HCs who underwent CTA for other reasons. Premature ACS was defined as ACS in men ≤55 years or women ≤65 years old. The study protocol was approved by the local ethics committee of the Beijing Friendship Hospital (2020-P2-008-01) and conformed to the tenets of the Declaration of Helsinki. All participants provided written informed consent.

Each participant underwent an ophthalmic examination, including best-corrected visual acuity, slit-lamp examination, intraocular pressure, fundus color photography, and optical coherence tomography. Patients with important ocular lesions, such as ocular refractive media turbidity, orbital space-occupying diseases, glaucoma, optic neuritis, or systemic diseases causing ocular fundus lesions, were excluded. The electronic medical records of all participants were reviewed to collect general information, laboratory parameters, echocardiography results, coronary angiography results, and concomitant medication.

### Ophthalmic artery three-dimensional reconstruction

**Figure 1:**
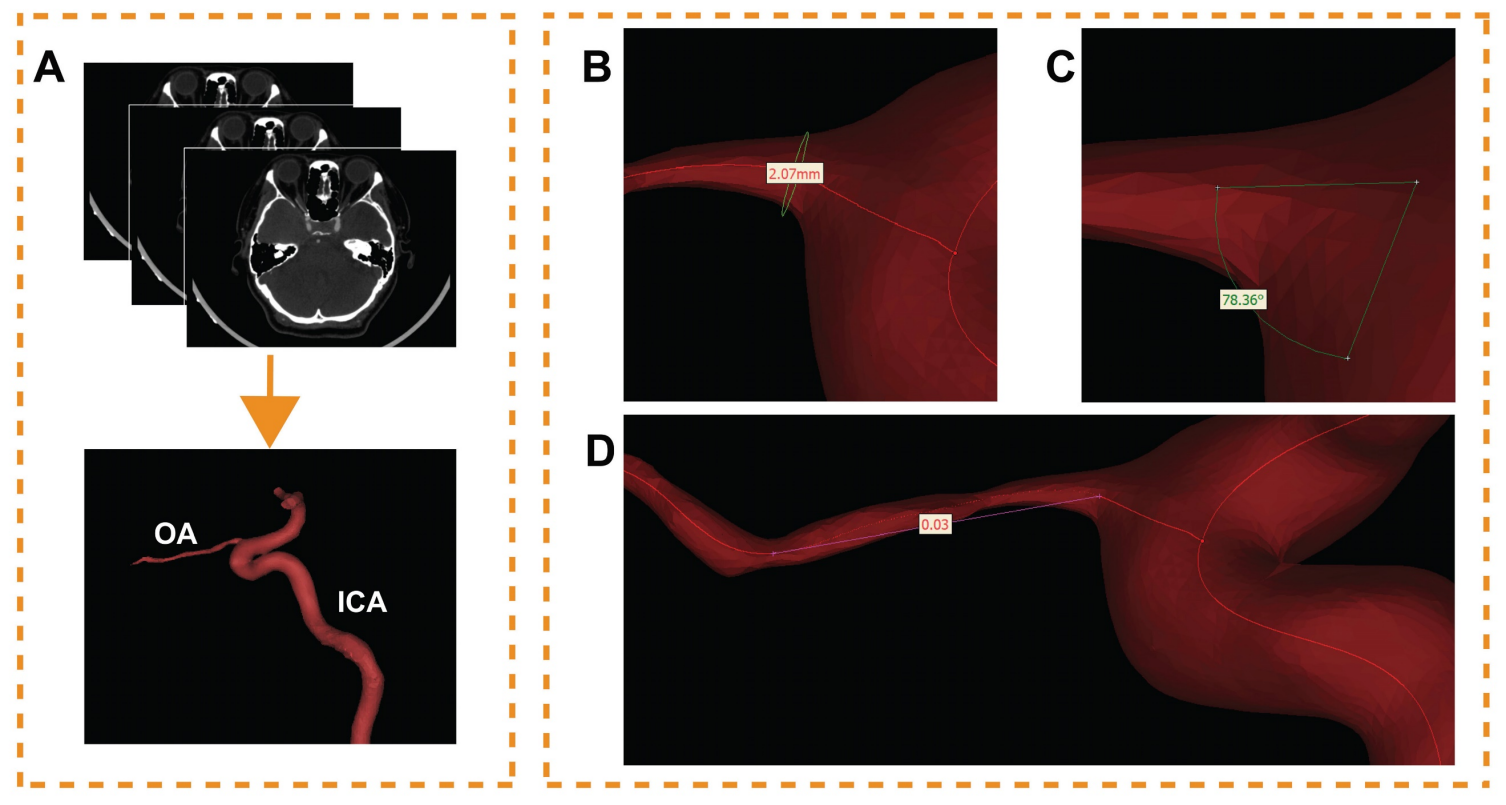
Ophthalmic artery (OA) three-dimensional reconstruction and morphological measurement. (A) The process of OA reconstruction; ICA, internal carotid artery. (B) The initial OA diameter measurement. (C) Angle measurement between OA and ICA. (D) Measurement of the OA tortuosity.

The CTA DICOM image was imported into Mimics 21.0 (Materialise, Ann Arbor, MI, USA). An image segmentation technique was used to reconstruct one of the OAs visible on the CTA image for each participant. During this process, model boundaries were manually edited to remove any adjacent structures that could potentially affect the accuracy of the OA reconstruction. These 'interfering structures' were defined as any anatomical elements not directly related to the blood flow within the OA, including but not limited to adjacent vessels, bone, soft tissue, and any artifacts present in the CTA images. Only the OA, and a specific segment of the ICA were included in the final model (Figure 1A). The preliminary OA model was calculated and imported into Geomagic Studio 14.0 (3D Systems, Rock Hill, SC, USA). After smoothing the model's surface, a solid blood vessel model was created.

Morphological data of the OA models were measured. The centerline of each model was generated; then, the best-fit diameter of the initial OA (Figure 1B), the angle between the OA and ICA centerline (Figure 1C) and the tortuosity of the OA (Figure 1D) were acquired. The initial OA was defined as the area where the OA originated from the ICA. The diameter and tortuosity of C4 and C5 segments of the ICA were measured by the same method. Two experienced ophthalmologists collected all the data.

### Hemodynamic numerical simulation

Based on our previous research^10^, a finite-volume method for steady flow was used in the hemodynamic numerical simulation using ANSYS Fluent 15.0 (ANSYS, Inc., Canonsburg, PA, USA). Each OA model was discretized into approximately 400,000 tetrahedral and tri-prism mixed elements using ANSYS ICEM CFD. The blood vessels were assumed to be rigid and non-slipped, and the simulated blood was considered to be a steady-state, laminar, incompressible Newtonian fluid. The governing equations for the numerical simulation were the Navier-Stokes equation and mass conservation equation:


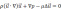

(1)


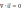

(2)

In the formula, 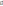 represents the velocity vector, 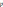 is the pressure, 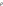 is the blood density, and μ is blood viscosity. The blood viscosity and density were set to 3.5 × 10^-3^ kg/ms and 1050 kg/m^3^, respectively. Based on the literature, we adopted a systolic and diastolic mean flow velocity of 0.34 m/s as the inlet velocity (velocity of the ICA siphon). For simplification, the outlet was set to a pressure boundary condition of P = 0 Pa. All models were set to the same boundary conditions.

The OA hemodynamic data were obtained using the Ansys Fluent post-processing software. The blood flow velocity, wall shear stress (WSS), and initial OA pressure were obtained quantitatively. The mass flow of the OA and ICA in flux reports was obtained. Additionally, the mass flow ratio, defined as the fraction of the ICA flowing into the OA, was calculated.

### Retinal and choroidal vessel information acquisition

The retinal vascular information was obtained from color fundus images (Kowa, Tokyo, Japan). Retinal vessel caliber (RVC) was measured by the Integrative Vessel Analysis (IVAN software, Australia), using a similar method to our previous study^11^. RVC included the following main parameters: central retinal artery equivalent (CRAE), central retinal vein equivalent (CRVE), and arteriole to venule ratio (AVR). Choroidal vascular data were obtained by enhanced depth imaging optical coherence tomography (EDI-OCT, Heidelberg Spectralis OCT, scan mode 10 mm). The CVI is luminal area divided by total choroidal area and was acquired using a similar method to the one mentioned by Agrawal *et al.*^12^. CVI in the whole choroid region and within 1500 μm of the central fovea was measured by EDI-OCT.

### Statistical analysis

Variables were tested for normality using the Shapiro-Wilk test. Data with normal distribution are expressed as mean ± standard deviation, while descriptive data with non-normal distribution are expressed as the median (25-75%). In multiple group comparisons, continuous variables with normal distribution were analyzed using one-way ANOVA, whereas variables with non-normal distribution were tested using the Kruskal-Wallis *H* test. The Bonferroni correction was used for multiple comparisons. Comparisons between two groups were performed using the *t*-test or Mann-Whitney *U* test, depending on normality. Categorical variables were expressed as numbers and percentages and analyzed using the *χ*^2^ or Fisher's exact test, as appropriate. Pearson's correlation coefficient and linear regression were used to determine the correlations between continuous variables, non-normally distributed variables were converted into natural logarithms. Statistical analyses were performed using SPSS Statistics 26.0 (IBM, Armonk, NY, USA).

## Results

### Baseline characteristics

In total, 116 OA models were reconstructed. Table 1 shows the baseline characteristics of the 116 participants (30 healthy controls [HCs]), 30 with premature ACS, and 56 with non-premature ACS). There were no significant differences for sex (*P* = 0.124) and type of ACS (*P* = 0.074,* P* = 0.877, *P* = 0.102, respectively). No differences in peripheral arterial disease (*P* = 0.173), history of ischemic stroke (*P* = 0.019, not significant after Bonferroni correction), or family history of coronary atherosclerotic heart disease (*P* = 0.104). A higher proportion of patients with ACS had a current smoking status, hypertension, diabetes, and dyslipidemia (*P* = 0.001, *P* <0.001, *P* = 0.005, and *P* = 0.010, respectively). The clinical, laboratory, echocardiographic, and medication details of the patients with ACS are shown in Table 2. Except for six participants in the premature ACS group, all patients with ACS underwent percutaneous coronary intervention (PCI) or coronary artery bypass grafting before CTA.

### Morphological analysis

We obtained the OA morphological data of all participants by measuring models (Table 3). The mean C5 segment diameter of the premature ACS group was significantly smaller than that in HCs (4.79 ± 0.77 vs. 5.10 ± 0.26 mm; *P* = 0.025). No significant differences were found among the three groups in the diameter, angle and tortuosity of the OA, the diameter of the C4 segment, and the tortuosity of the C4-C5 segment.

### Hemodynamic analysis

**Figure 2:**
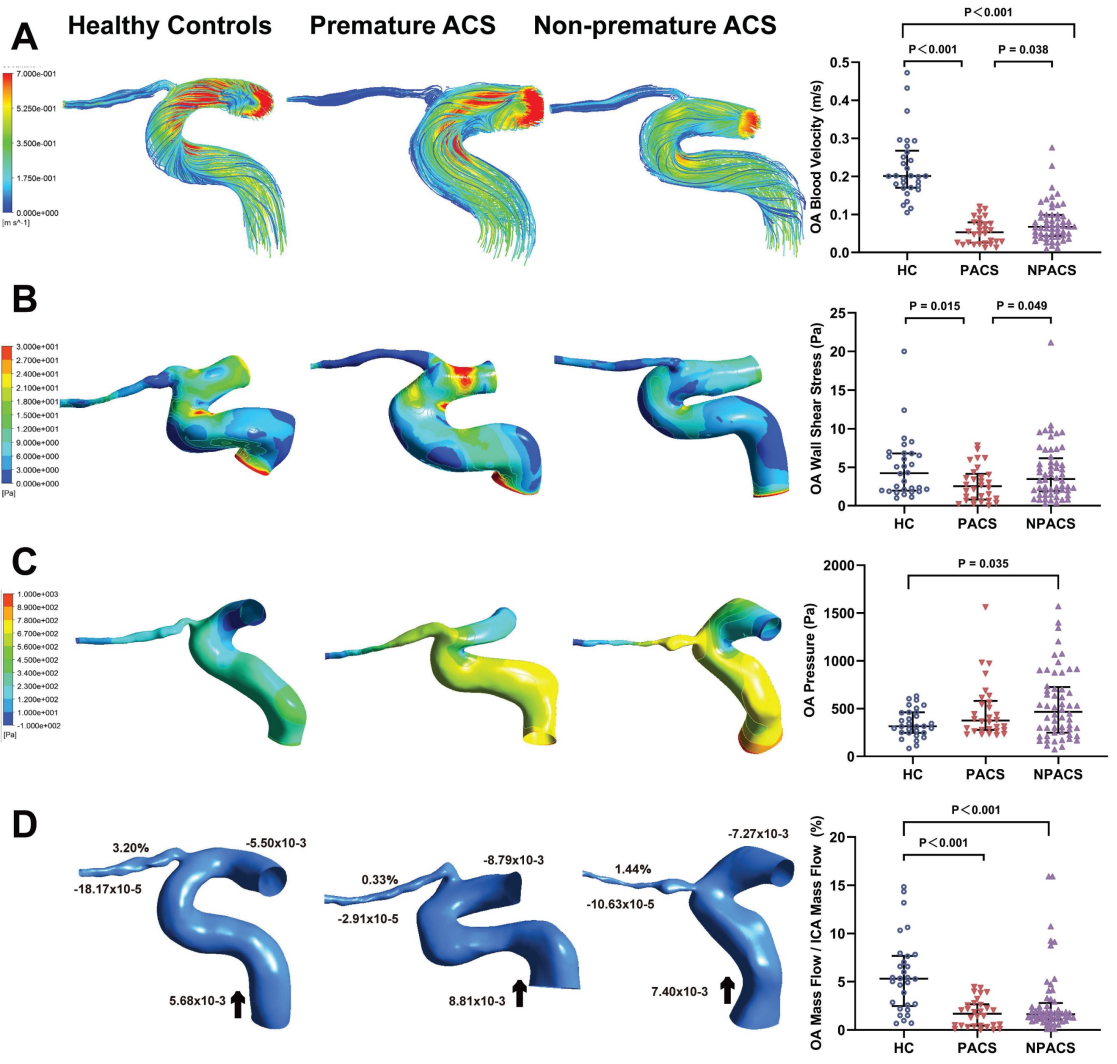
Hemodynamics characteristics of ophthalmic artery (OA). (A) The streamlines are colored according to the magnitude of velocity in three typical patients; Comparison of OA blood velocity. (B) The wall shear stress contour of OA in three typical patients; Comparison of OA wall shear stress. (C) The pressure contour of OA in three representative models; Comparison of OA pressure. (D) Mass flow (kg/s) and mass flow ratio of the OA to ipsilateral internal carotid artery (%) of three representative models; Comparison of mass flow ratio of OA to ipsilateral internal carotid artery (%); Inlet (+), outlet (-), Blood flow direction (black arrow). ACS, acute coronary syndrome; HC, healthy controls; PACS, premature ACS; NPACS, non- premature ACS.

Table 3 shows the quantitative measurement results of the initial OA hemodynamics. Streamline charts of each OA model were drawn according to the numerical simulation (Figure 2A). The colors in the streamlined charts represent the different blood flow velocities. Streamlines near red indicate higher speeds. The blood flow velocities of the OA in all disease groups were lower than those of the control group (*P* < 0.001). Moreover, the premature ACS group had slower OA blood velocities than the non-premature ACS group (*P* = 0.038).

Figure 2B shows the contour charts of the WSS. The WSS of the initial OA in patients with premature ACS was significantly lower than that in HCs (*P* = 0.015) and in patients with non-premature ACS (*P* = 0.049). The non-premature ACS group had a higher OA pressure than the control group (*P* = 0.035) (Figure 2C).

Similarly, we obtained mass flow data for each OA model (Figure 2D). The mass flow ratios in all ACS groups were lower than those in the control group (*P* <0.001). Nevertheless, no significant difference was found between participants with premature ACS and those with non-premature ACS (*P* = 0.328).

### Retinal and choroidal vascular analysis

**Figure 3:**
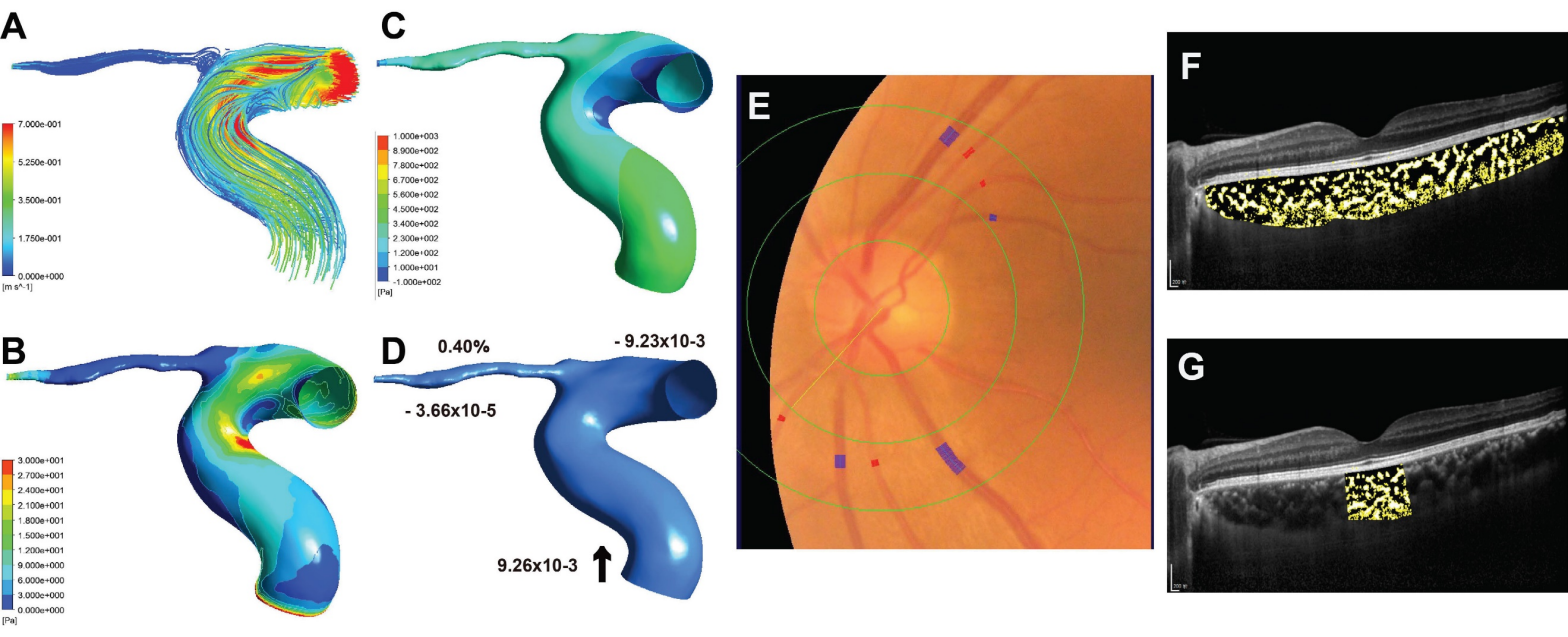
Sample case (left eye) for premature ACS group. (A) The streamlines are colored according to the magnitude of the ophthalmic artery (OA) velocity. (B) The wall shear stress contour of OA. (C) The pressure contour of OA. (D) Mass flow (kg/s) and mass flow ratio of the OA to ipsilateral internal carotid artery (%); Inlet (+), outlet (-), Blood flow direction (black arrow). (E) Quantitative analysis of retinal vessel caliber by Integrative Vessel Analysis (IVAN software, Australia); veins are identified in purple and arteries in red; in this case, the central retinal artery equivalent value is 125.89mm, central retinal vein equivalent value is 224.34mm, and arteriole to venule ratio value is 0.56. (F) Choroidal vascularity index (CVI) in the whole choroid region; total choroidal area (TCA) and luminal area (LA) were measured using a binarized image; overlay image of enhanced depth imaging optical coherence tomography scan with region of interest obtained after image binarization; yellow pixels represent stroma and black pixel represent vascular lumen; CVI was determined by dividing TCA by LA; in this case, the CVI value is 0.31. (G) CVI within 1500μm of central fovea is 0.33.

**Figure 4:**
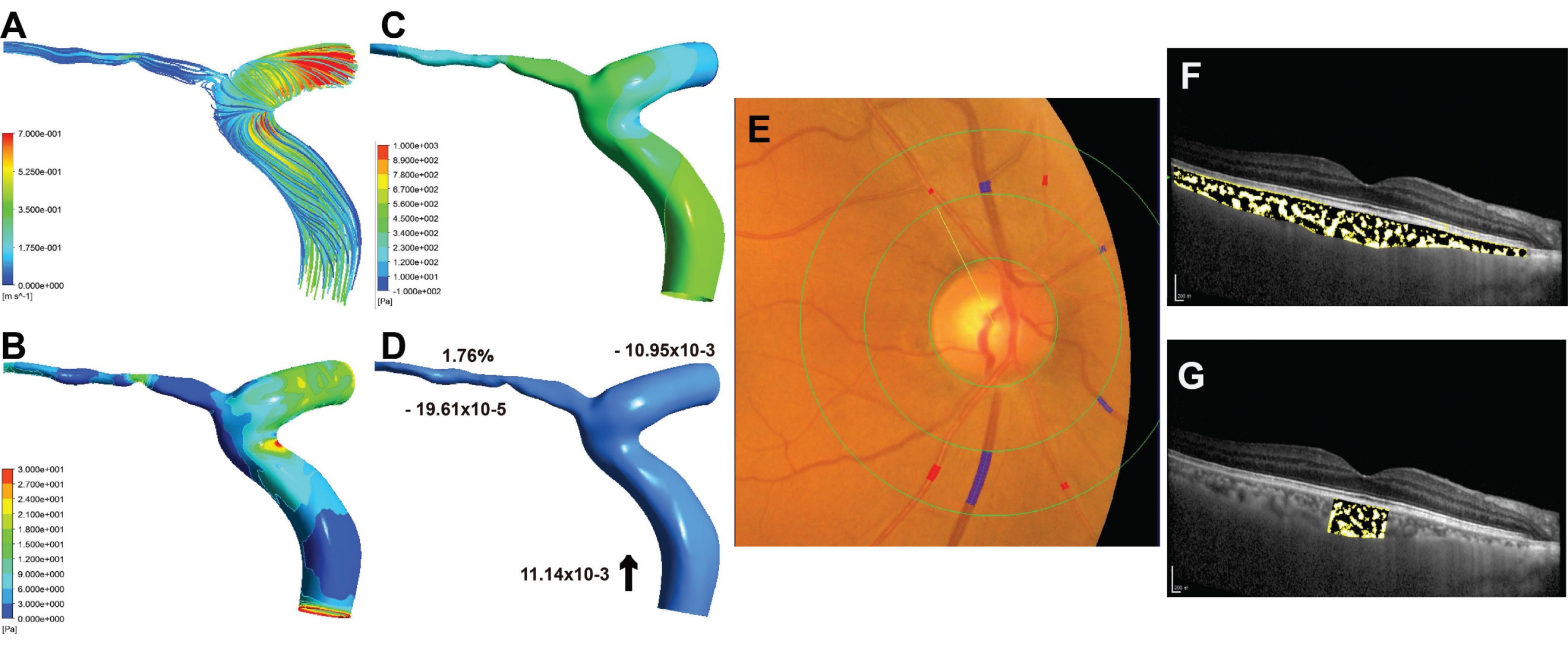
Sample case (right eye) for non-premature ACS group. (A) The streamlines of the ophthalmic artery (OA). (B) The wall shear stress contour of the OA. (C) The pressure contour of the OA. (D) Mass flow (kg/s) and mass flow ratio of the OA to ipsilateral internal carotid artery (%); Inlet (+), outlet (-), Blood flow direction (black arrow). (E) Quantitative analysis of retinal vessel caliber by Integrative Vessel Analysis (IVAN software, Australia); veins are identified in purple and arteries in red; in this case, the central retinal artery equivalent value is 134.58mm, central retinal vein equivalent value is 226.67mm, and arteriole to venule ratio value is 0.59. (F) Choroidal vascularity index (CVI) in the whole choroid region; total choroidal area (TCA) and luminal area (LA) were measured using a binarized image; overlay image of enhanced depth imaging optical coherence tomography scan with region of interest obtained after image binarization; yellow pixels represent stroma and black pixel represent vascular lumen; CVI was determined by dividing TCA by LA; in this case, the CVI value is 0.38. (G) CVI within 1500μm of central fovea is 0.34.

Some eyes in participants with premature ACS showed smaller CRAE than those of the participants with non-premature ACS (Figure 3E, Figure 4E), and the AVR was correspondingly smaller. Similarly, in these cases, patients with premature ACS also had smaller choroidal vascularity index (CVI) values than those with non-premature ACS (Figure 3F, 3G; Figure 4F, 4G).

### Correlation analysis

**Figure 5:**
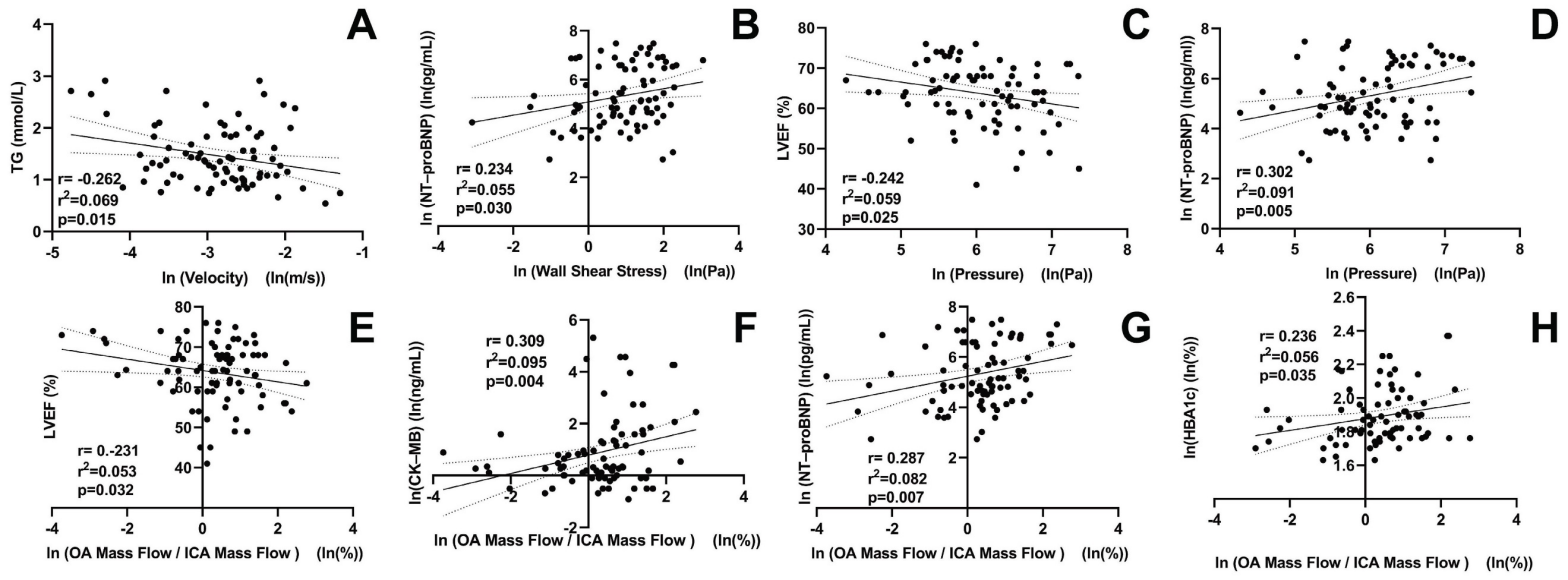
Correlation between ophthalmic artery (OA) hemodynamic variables and clinical parameters of patients with ACS. TG, triacylglycerol; NT-proBNP, N-terminal pro-B-type natriuretic peptide; LVEF, left-ventricular ejection fraction; CK-MB, creatine kinase isoenzyme-MB; HBA1c, hemoglobin A1c; ICA, internal carotid artery; ln, natural log of the variable.

Figure 5 shows the correlations between OA characteristics and clinical parameters. OA blood flow velocity was negatively correlated with triacylglycerol levels (*r* = -0.262,* P* = 0.015). The OA WSS was positively correlated with N-terminal pro-B-type natriuretic peptide (*r* = 0.234,* P* = 0.030). OA pressure was positively correlated with N-terminal pro-B-type natriuretic peptide (*r* = 0.302,* P* = 0.005), but negatively correlated with left ventricular ejection fraction (*r* = -0.242,* P* = 0.025). The mass flow ratios of the OA to the ipsilateral ICA were positively correlated with creatine kinase isoenzyme-MB (*r* = 0.309,* P* = 0.004), N-terminal pro-B-type natriuretic peptide (*r* = 0.287,* P* = 0.007), and hemoglobin A1c levels (*r* = 0.236,* P* = 0.035). In contrast, it was negatively correlated with left-ventricular ejection fraction (*r* = -0.231,* P* = 0.032).

## Discussion

This study demonstrated the characteristics of the ocular hemodynamics in patients with premature ACS based on CFD numerical simulations. In patients with premature ACS, OA blood flow velocity, WSS and mass flow were decreased, and retinal and choroid microcirculation hemodynamics were changed. Most studies have focused on the correlation between retinal vascular morphology and ACS^13^-^15^, and hemodynamic data were few. Color Doppler imaging (CDI) was used for most measurements. Wu *et al.* found that central retinal artery blood flow velocity decreased and resistance increased in patients with coronary heart disease. Additionally, they found that retinal artery blood flow was associated with endothelial dysfunction^16^, which supports the results of this study. However, CDI has limited ability to observe OA. The numerical simulation based on CFD solves the difficult problem of the OA observation and measurement, and provides an effective means to explore the relationship between OA and ischemic heart disease.

Consistent with our previous study^10^, the initial OA blood velocity was lower in patients with ACS than in HCs. We compared the OA blood flow velocity of STEMI, NSTEMI, and UA in ACS and found no significant differences. Furthermore, we found that patients with premature ACS had lower blood flow velocities than those with non-premature ACS. Premature ACS may differ from non-premature ACS in pathogenesis and prognosis and may also have different effects on systemic hemodynamics. Reduced blood flow velocities were found in carotid and middle cerebral arteries in patients with coronary slow flow phenomenon, and patients presented with systemic arteriosclerosis simultaneously^17^. The pathogenesis of arteriosclerosis involves endothelial dysfunction, lipid deposition, oxidative stress, and other aspects, but the role of hemodynamics in the pathogenesis and development of arteriosclerosis should not be ignored. The main factor in plaque formation is the reaction time between molecules and the surface, while slow blood flow increases the residence time of blood passing through the artery, which increases the likelihood of blood particles reacting with wall vessels^18^. Therefore, the OA in patients with premature ACS is more likely to develop atherosclerosis. In addition, there is evidence that the coronary slow flow phenomenon has important prognostic significance in patients with chest pain and angina diagnosed with normal coronary angiography^19^. Low coronary blood flow has been associated with myocardial ischemia, life-threatening arrhythmias, sudden cardiac death, and even increased cardiovascular mortality^17^.

This study used CFD to reveal WSS changes in OA. The WSS is an important biomechanical indicator associated with many physiological and pathological phenomena in the cardiovascular system. WSS can affect the occurrence and development of atherosclerotic plaques by regulating the function of vascular endothelial cells^20^. The flowing blood exerts friction on endothelial cells in the walls of blood vessels and acts as a sensor for WSS. Endothelial cells respond by changing their morphology, function, and gene expression to transmit WSS signals into the cell interior, thus maintaining normal blood flow, regulating vascular diameter, and maintaining vascular system homeostasis^21^. There is increasing evidence that atherosclerotic lesions preferentially arise in areas of flow disturbance associated with low shear stress^22^. In addition, low WSS accelerates endothelial turnover, leading to increased lipid uptake and promoting plaque necrotic core formation^23^. Given the OA as the primary blood supply to the eye, fluctuations in WSS could significantly impact ocular microcirculation. A previous study has indicated that changes in the OA may precede those in the retinal blood vessels in patients with ocular ischemic syndrome^24^. Reduced WSS within the OA may correlate with the initial stages of arterial remodeling and a decrease in blood flow, potentially preceding any noticeable alterations in the retinal circulation. Through hemodynamic simulation, we found that the WSS of the OA was lower in patients with premature ACS, whereas this change did not exist in patients with non-premature ACS. This implies that more attention should be paid to the ocular conditions of male and female patients with premature ACS who are aged ≤ 55 and ≤ 65 years, respectively, because they may be more prone to arterial stenosis or obstructive ocular complications. This may also explain the predisposition of patients to arterial obstructive disease after ACS.

Qiao *et al.* studied nonlinear pulsating flow in S-shaped arteries using finite element methods. Numerical simulation results showed that the wall pressure in the curved artery changes dramatically, especially in larger models^25^. We performed the measurement of tortuosity to exclude the influence of the curved shape of the ICA and OA on the simulation results. Research indicates that patients with ACS are at a significantly higher likelihood of developing retinal microaneurysms and dot bleeding compared to individuals with stable coronary artery disease^14^. The elevated OA pressure in the non-premature ACS group in our study could indicate a propensity for increased vascular pressure and potential dilation, which may predispose these individuals to the development of microaneurysms and OA aneurysm. Blood flow dynamics are instrumental in aneurysm development, influencing the collagen remodeling of the aneurysmal wall^26^. In terms of blood flow, this study found that both premature and non-premature ACS had reduced OA mass flow ratio. Ocular ischemia can cause a variety of eye diseases; hence, patients with a history of ACS should be alert to the occurrence of ischemia-related ocular diseases. Our observation of retinal and choroidal vessels showed that patients with premature ACS had smaller CRAE and CVI. Due to insufficient blood supply of OA, retinal blood perfusion and perfusion pressure decreased. Once the drop of ocular perfusion pressure exceeds the compensatory limit of retinal vessels, the retinal arterioles will shrink^27^. In addition, decreases in CVI have been observed in a variety of ocular diseases. Studies showed a significant reduction of CVI in patients with age-related macular degeneration^28^. Decreased choroidal vessels suggest decreased perfusion, which may lead to choroidal neovascularization. Moreover, progression of diabetic retinopathy is characterized by a continuous decrease in CVI, which is inversely correlated^29^. The hemodynamic environment may change before the morphology of OA changes, affecting the downstream microcirculation of the retina and choroid. Early ischemic lesions of the retina and choroid may occur in patients with premature ACS more than in patients with non-premature ACS.

Our study found significant correlations between OA's hemodynamic parameters and key clinical indicators in patients with ACS. Elevated lipids and higher fasting blood glucose levels have been reported more commonly in premature ACS compared to non-premature ACS^30^, ^31^. Additionally, higher levels of triglycerides were related to the risk of premature ACS^32^. This could explain why triglyceride levels were negatively correlated with OA blood flow velocity in this study. The association of N-terminal pro-B-type natriuretic peptide (NT-proBNP) with multiple OA hemodynamic parameters is noteworthy. NT-proBNP is closely associated with the extent of myocardial ischemia and independently associated with an increased risk of major adverse cardiovascular events in patients with ACS^33^, ^34^. Elevated NT-proBNP has been significantly associated with poor clinical outcomes in patients with ACS who have undergone successful percutaneous coronary intervention (PCI) and have a normal left ventricular ejection fraction^35^. Considering the autoregulation mechanisms of ocular blood flow^27^, chronic blood flow disorders may lead to chronic changes or impaired vascular regulation, further causing lesions. Therefore, changes in OA hemodynamics could provide a new direction for the early prediction and prognosis of premature ACS. Future research with longitudinal data is needed to further explore the predictive value of OA hemodynamic parameters for ACS outcomes.

This study had some limitations. First, the layer thickness of the CTA scan limited the accuracy of the three-dimensional reconstruction. Second, due to the lack of high-resolution dynamic imaging data, steady-state CFD simulations were performed, which could not reflect the instantaneous hemodynamic changes of the OA. Furthermore, because of the lack of patient-specific measurements, we set the same boundary conditions for all groups. Future research needs to combine clinical data to increase individualized boundary conditions and increase sample size to improve results integrity. In future work, the relationship between ocular conditions and systemic indicators in patients with premature ACS should be further explored. More ocular examinations, such as CDI, should be incorporated for further analysis and quantitative verification. The application of ocular multimodal imaging in CFD simulation would be an interesting exploration in the future.

## Conclusions

This study revealed that patients with premature ACS had slower OA blood flow velocity and lower WSS, which increased the risk of plaque formation. Changes of hemodynamics of the OA and microcirculation of the retina and choroid in patients with premature ACS appeared before OA morphological changes. Moreover, premature ACS may aggravate ocular ischemic lesions more than non-premature ACS. Our findings could potentially guide future studies into a better understanding of the association of ocular lesions with systemic conditions in patients with premature ACS.

## Figures and Tables

**Table 1:** Baseline Characteristics of the Participants

Variables	HC (n=30)	Premature ACS (n=30)	Non-premature ACS (n=56)	*P* value
Age (y), mean ± SD	62.13±5.18	55.07±6.53	67.61±4.38	**<0.001**
Female sex, n (%)	14 (47)	9 (30)	11 (20)	**0.032**
STEMI, n (%)	-	2 (7)	13 (23)	0.074
NSTEMI, n (%)	-	6 (20)	12 (21)	0.877
UA, n (%)	-	22 (73)	31 (55)	0.102
Current smoking, n (%)	6 (20)	15 (50)	38 (69)	**<0.001**
Hypertension, n (%)	18 (60)	27 (90)	49 (88)	**0.003**
Diabetes mellitus, n (%)	4 (13)	20 (67)	31 (55)	**<0.001**
Dyslipidaemia, n (%)	16 (53)	24 (80)	46 (82)	**0.010**
PAD, n (%)	4 (13)	10 (33)	12 (21)	0.173
History of ischemic stroke, n (%)	2 (7)	9 (30)	19 (34)	0.019
Family history of CAD, n (%)	3 (10)	7 (23)	17 (30)	0.104

HC, healthy controls; ACS, acute coronary syndrome; STEMI, ST-segment elevation myocardial infarction; NSTEMI, non-STEMI; UA, unstable angina; PAD, peripheral arterial disease; CAD, coronary atherosclerotic heart disease. Bonferroni correction was used for multiple comparisons. Bold values are significant.

**Table 2:** Baseline Characteristics of Patients with Premature ACS and Non-premature ACS

Variables	Premature ACS (n=30)	Non-premature ACS (n=56)	*P* value
**Clinical characteristics**			
BMI (kg/m2), mean ± SD	26.81±3.00	25.78±2.84	0.119
DAC (cm), mean ± SD	96.20±11.64	90.54±10.11	**0.021**
Heart rate (bpm), mean ± SD	74.47±9.72	72.80±12.57	0.530
Systolic BP (mmHg), mean ± SD	132.47±16.38	135.14±23.73	0.583
Diastolic BP (mmHg), mean ± SD	82.63±10.56	76.55±17.81	0.091
**Laboratory parameters**			
TnI (ng/mL), median (IQR 25%-75%)	0.01 (0.001-0.04)	0.03 (0.004-0.86)	0.055
TnT (ng/mL), median (IQR 25%-75%)	0.01 (0.01-0.02)	0.02 (0.01-0.13)	**0.002**
CK (U/L), median (IQR 25%-75%)	94.00 (62.00-120.50)	117.00 (70.00-227.00)	0.106
CK-MB (ng/mL), median (IQR 25%-75%)	0.90 (0.60-1.60)	1.70 (1.10-4.90)	**0.003**
LDH (U/L), median (IQR 25%-75%)	176.00 (145.00-185.50)	194.00 (153.00-236.00)	**0.045**
NT-proBNP (pg/mL), median (IQR 25%-75%)	70.00 (47.45-218.50)	294.00 (103.00-967.00)	**0.002**
Scr (μmol/L), mean ± SD	71.63±13.33	67.83±13.13	0.207
FBG (mmol/L), median (IQR 25%-75%)	6.16 (5.10-8.26)	6.40 (5.90-8.60)	0.103
HBA1c (%), median (IQR 25%-75%)	6.10 (5.70-7.30)	6.60 (6.00-7.40)	0.350
TC (mmol/L), mean ± SD	4.04±0.99	3.82±1.02	0.350
TG (mmol/L), mean ± SD	1.48±0.54	1.45±0.59	0.820
HDL (mmol/L), mean ± SD	1.14±0.49	1.00±0.24	0.068
LDL (mmol/L), mean ± SD	2.22±0.72	2.16±0.76	0.720
Sodium (mmol/L), mean ± SD	140.24±1.39	140.10±2.55	0.778
Potassium (mmol/L), mean ± SD	4.00±0.30	3.93±0.38	0.429
TyG index, mean ± SD	7.29±0.53	7.34±0.53	0.665
**Echocardiography, mean ± SD**			
LVEF (%)	66.05±4.51	62.43±8.26	**0.029**
E/A	0.89±0.28	0.82±0.22	0.261
Cardiac index (L/min/m2)	2.66±0.45	2.85±0.54	0.133
**Coronary artery lesions, n (%)**			
Single vessel lesion	2 (7)	10 (18)	0.153
Multiple vessel lesions	28 (93)	46 (82)	0.153
**Concomitant medication, n (%)**			
Statin	28 (93)	46 (82)	0.153
Aspirin	26 (87)	47 (84)	0.735
Clopidogrel/ Ticagrelor	21 (70)	44 (79)	0.378
ACE inhibitor/ARB	12 (40)	24 (43)	0.798
Beta blocker	20 (67)	36 (64)	0.825
Calcium channel blocker	11 (37)	20 (36)	0.930
Insulin	1 (3)	7 (13)	0.163

BMI, body mass index; BP, blood pressure; TnI, troponin I; IQR, interquartile range; TnT, troponin T; CK, creatine kinase; CK-MB, creatine kinase isoenzyme-MB; LDH, lactate dehydrogenase; NT-proBNP, N-terminal pro-B-type natriuretic peptide; Scr, serum creatinine; FBG, fasting blood glucose;HBA1c, hemoglobin A1c; TC, total cholesterol; TG, triacylglycerol; HDL, high-density protein; LDL, low-density protein; TyG, triglyceride glucose index, calculated as the ln [fasting triglycerides (mg/dL)×fasting plasma glucose (mg/dL)/2]; LVEF, left-ventricular ejection fraction; E/A, ratio of early to late transmitral flow velocity; ACE, angiotensin-converting enzyme; ARB, angiotensin receptor blocker. *P* < 0.05 is significant (bold values).

**Table 3:** Quantitative measurement of morphological and hemodynamic parameters

Variables	HC (n=30)	Premature ACS (n=30)	Non-premature ACS (n=56)	*P* value
**Morphological analysis, mean ± SD**				
OA diameter, mm	2.14±0.39	2.30±0.41	2.28±0.45	0.265
OA tortuosity	0.04±0.02	0.04±0.02	0.03±0.02	0.125
Angle, °	73.51±14.30	77.66±10.14	73.96±12.53	0.345
C4 diameter, mm	5.60±0.39	5.43±0.35	5.54±0.41	0.253
C5 diameter, mm	5.10±0.26	4.79±0.77	4.97±0.47	0.077^a^
C4-C5 tortuosity	0.28±0.08	0.27±0.08	0.26±0.07	0.569
**Hemodynamic analysis, median (IQR 25%-75%)**				
Velocity, m/s	0.20 (0.17-0.27)	0.05 (0.03-0.08)	0.07 (0.04-0.10)	**<0.001** ^b^
WSS, Pa	4.22 (1.94-6.79)	2.51 (0.79-4.14)	3.44 (1.87-6.17)	0.043^c^
Pressure, Pa	314.63 (246.66-462.00)	375.37 (276.82-579.39)	466.68 (248.80-724.97)	0.100^d^
Mass flow ratio, %	5.31 (2.49-7.68)	1.69 (0.44-2.67)	1.63 (1.10-2.80)	**<0.001**

HC, healthy controls; ACS, acute coronary syndrome; OA, ophthalmic artery; WSS, wall shear stress; Bonferroni correction was used for multiple comparisons. Bold values are significant. ^a^, a significant difference in C5 diameter between HC and premature ACS group (*P* = 0.025); ^b^, a significant difference in velocity between premature ACS and non-premature ACS group (*P* = 0.038); ^c^, WSS in premature ACS group was significantly lower than that in HC (*P* = 0.015) and in non-premature ACS (*P* = 0.049); ^d^, non-premature ACS group had a higher OA pressure than HC (*P* = 0.035).

## Abbreviations

ACS: acute coronary syndrome
AVR: arteriole to venule ratio
CDI: color Doppler imaging
CFD: computational fluid dynamics
CRAE: central retinal artery equivalent
CRVE: central retinal vein equivalent
CTA: computed tomography angiography
CVI: choroidal vascularity index
HC: healthy controls
ICA: internal carotid artery
NSTEMI: non-ST-segment elevation myocardial infarction
OA: ophthalmic artery
PCI: percutaneous coronary intervention
RVC: retinal vessel caliber
STEMI: ST-segment elevation myocardial infarction
UA: unstable angina
WSS: wall shear stress
